# Skin perfusion pressure in lower extremities at haemodialysis initiation is associated with 1-year mortality and cardiovascular disease

**DOI:** 10.1007/s10157-025-02680-1

**Published:** 2025-04-23

**Authors:** Yoshifumi Hamasaki, Mikie Imafuku, Kana Suzuki, Shutaro Ishii, Ryo Matsuura, Daisuke Yamada, Masaomi Nangaku

**Affiliations:** 1 Department of Hemodialysis and Apheresis, The University of Tokyo Hospital, 7-3-1 Hongo, Bunkyo-Ku, Tokyo, 113-8655 Japan; 2 Department of Nephrology and Endocrinology, The University of Tokyo Hospital, Tokyo, Japan

**Keywords:** Skin perfusion pressure (SPP), Haemodialysis, Dialysis nurse, Peripheral arterial disease (PAD)

## Abstract

**Background:**

Skin perfusion pressure (SPP) is a noninvasively obtained and useful measurement for assessing peripheral arterial disease (PAD). Decreased SPP in lower extremities is associated with poor survival in maintenance haemodialysis (HD) patients. Nevertheless, the prognostic significance of SPP at HD initiation has not been determined. We investigated the relation between SPP and death or cardiovascular disease (CVD) in incident HD patients.

**Methods:**

Data were collected retrospectively from patients with SPP measurements taken by dialysis nurses at HD initiation during 2020–2023. Then we assessed the association between the minimum value of SPP in the bilateral dorsal/plantar portions of each patient (SPPmin) and outcomes, consisting of mortality and CVDs within 1 year after HD initiation.

**Results:**

This study examined 104 incident HD patients with a median age of 74 (79% male). Based on the suggested cut-off value from receiver operating characteristic analysis, patients were divided into two groups: patients with SPPmin ≥ 60 mmHg (higher SPPmin) and those with SPPmin < 60 mmHg (lower SPPmin). Kaplan–Meier analysis indicated the 1-year survival and CVD-free rate as significantly lower in the lower SPPmin group than in the higher SPPmin group (*p* < .001). Cox proportional hazards analyses showed lower SPPmin as associated with the composite outcome. The relation between lower SPPmin and outcome held true in each subgroup with cardiovascular risk such as older age and history of CVD.

**Conclusion:**

SPP measured by dialysis nurses at HD initiation is associated with 1-year adverse outcomes in incident HD patients.

**Supplementary Information:**

The online version contains supplementary material available at 10.1007/s10157-025-02680-1.

## Introduction

Peripheral arterial disease (PAD) in the lower extremities is a commonly occurring complication in patients with chronic kidney disease (CKD) and end-stage kidney disease (ESKD). Reportedly, CKD is a risk factor for PAD: up to 20–25% and 25–45% of non-dialysis and ESKD patients, respectively, have PAD [1–4]. Because PAD is one manifestation of polyvascular diseases accelerated by endothelial dysfunction and vascular calcification, PAD is associated strongly with mortality and incidence of systemic cardiovascular diseases (CVDs) in CKD and ESKD patients [1, 2, 5–9]. In a recent study, comparing patients with eGFR ≥ 60 mL/min/1.73 m^2^ and no PAD, dialysis patients with PAD had 12-fold, 2.7-fold, and 1.9-fold higher risks, respectively, for lower-limb complications, cardiovascular events, and mortality [6]. Early assessment of PAD is crucially important for stratifying the risk of adverse outcomes and for providing appropriate treatment and care for these high-risk patients.

To detect PAD, including asymptomatic cases, and to assess its severity, several non-invasive examinations are performed. Although ankle-brachial index (ABI) is recommended as the primary screening test for PAD in general, its diagnostic ability for PAD is limited in ESKD patients with advanced arterial calcification of the lower limb arteries [1]. The sensitivity of ABI to detect PAD was reported as only 29.9% in maintenance haemodialysis (HD) patients [4].

Skin perfusion pressure (SPP) measurement is a non-invasive examination for functional assessment of local microcirculation using laser Doppler [10]. SPP is anticipated for use in identifying PAD in dialysis patients because it is not influenced by arterial calcification. Several studies have demonstrated the capability of SPP for detecting PAD in HD patients: 50 mmHg was proposed as the cut-off value for SPP to detect PAD in maintenance HD patients, with sensitivity of 84.9% and specificity of 76.9% [4, 11, 12]. In ESKD patients, SPP has been reported to be associated with adverse clinical outcomes such as mortality or cardiovascular event [11, 13–15]. Routinely at our facility, dialysis nurses have assessed lower extremity blood flow by SPP during HD sessions for medical and nursing applications.

The usefulness of SPP and its relation to adverse outcomes in maintenance HD patients have been reported. Nevertheless, little is known about the relation between SPP at dialysis initiation and prognosis. This study was conducted to investigate the relation between SPP at HD initiation and adverse outcomes including mortality and new-onset CVDs within one year.

## Materials and methods

### Study design and patients

We conducted a retrospective observational cohort study including adult (age 18 years and older) patients initiating HD between May 1, 2020 and June 30, 2023 in a tertiary care hospital. All patients included in this study were patients inducted into conventional HD under inpatient care according to our usual clinical practice policy. To continue HD treatment, patients went to other outpatient HD clinics after discharge from our hospital or were transferred to another hospital when discharge to home was difficult. Exclusion criteria were the following: (1) lost to follow-up within 3 months after HD initiation, (2) patients without SPP data at HD initiation period, (3) transition from peritoneal dialysis, and (4) patients who needed HD temporary but who were weaned off it successfully before discharge from our hospital. Written informed consent was waived because of the characteristics of the retrospective observational study, but opt-out consent was adopted. We provided participants with both information of the study (purpose, method, required data, and duration) on a website and the opportunity for opting out. This study, conducted in accordance with the principles outlined in the Declaration of Helsinki, was approved by the Institutional Review Board of our institution.

### Data collection

Basic information of the participants was obtained from their electronic medical records: age, gender, cause of ESKD, history of smoking (current, or ex-smoker), history of diabetes, and history of CVD before HD initiation. Their data of body mass index (BMI) and systolic and diastolic blood pressure at the start of the HD session in which SPP was measured were obtained from electronic medical records. Data of ultrafiltration volume and intradialytic hypotension, defined as any symptomatic decrease in systolic blood pressure or a nadir intradialytic systolic blood pressure less than 90 mmHg, during the HD session with SPP measurement were also obtained [16]. Activities of daily living (ADL) were assessed based on the Barthel index, which had been evaluated by nurses at dialysis initiation. Laboratory data from immediately before HD initiation were also obtained from electronic medical records: haemoglobin (Hb), albumin (Alb), blood urea nitrogen (BUN), serum creatinine (Cr), corrected calcium, phosphate, uric acid, C-reactive protein (CRP), and total cholesterol (T.chol). The geriatric nutritional risk index (GNRI) at HD initiation was calculated using data of Alb, body weight, and ideal body weight according to methods described in the literature [17].

### Skin perfusion pressure (SPP) measurement

Measurement of SPP was performed using a system to monitor microcirculatory blood flow in the skin (Nahri MV monitor; Nexis Co. Ltd., Tokyo, Japan). Briefly, an optical sensor using laser Doppler technology was located on the measurement site. A cuff was wrapped over it. After the cuff was inflated over the supra-systolic pressure to shut off blood flow, it was depressurized gradually. The cuff pressure at which blood perfusion begins to be detected again, the inflection point of the cuff pressure curve, was defined as the SPP.

The SPP measurements were taken during the HD session (approximately 1 h after starting HD). Dehydration by HD was continued during SPP measurement. When patients had severe oedema in the lower extremities at HD initiation, SPP measurement was performed at the later HD session after the oedema was resolved. Trained dialysis nurses examined SPP at bilateral dorsal and plantar portions of feet for a patient lying supine on an HD bed at 24 °C room temperature. The inflection point of the cuff pressure curve, SPP, was identified automatically and was subsequently confirmed by two trained dialysis nurses. The minimum SPP values obtained from measurements at four locations in each patient were defined as SPPmin. SPP was not measured in patients with open wounds or symptomatic ischemic lesions at or distal to the measurement site.

### Outcomes

The primary outcomes were the composite of all-cause death and any CVD occurred within one year after HD initiation. The data of occurrence and date of the first observed outcome for each patient after HD initiation were obtained through electronic medical records. Patient survival was censored at kidney transplantation or at the date of the last follow-up.

### Statistical analysis

All statistical analyses were conducted using software (BellCurve for Excel; Social Survey Research Information Co., Ltd., Tokyo, Japan). Continuous data are expressed as mean ± standard deviation or median (interquartile range). Student *t*-tests and Mann–Whitney *U*-tests were used to compare continuous variables between two groups. Chi-square test and Fisher’s exact test were used to compare categorical variables. Spearman’s correlation test was applied to evaluate the correlation between clinical parameters. The Kaplan–Meier method and the log-rank test were used to compare differences in primary outcome between the groups divided according to SPP. Univariate and multivariate Cox proportional hazards regression analyses were performed to assess whether a target factor is associated with the primary outcome. Receiver operating characteristic (ROC) analysis was performed to evaluate the predictive ability of SPP for death or onset of CVDs within 1 year after HD initiation. Results for which a* p* value less than 0.05 was obtained were inferred as representing significant differences.

## Results

### Study participant characteristics

**Fig. 1:**
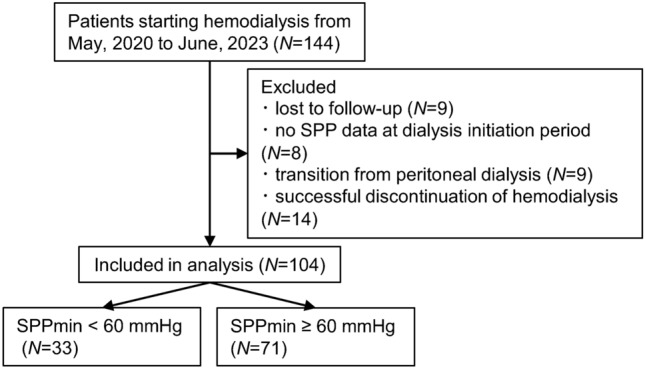
Flow diagram of inclusion, exclusion, and grouping of the study participants

Of the 144 patients who started HD during the study period, excluding those who met the exclusion criteria, 104 patients were included in the analysis (Fig. 1). Table 1 shows the patient characteristics and laboratory data. The median (IQR) age was 74 (61, 81) years old; 82 (79%) were male. The most common cause of ESKD was diabetic kidney disease (*n* = 37, 36%) followed by nephrosclerosis (*n* = 24, 23%). The mean value of SPPmin was 69 mmHg. The median (IQR) duration from HD initiation to SPP examination was 0 (0, 3) days. The primary outcome was observed in 15 (15%) patients, of whom 5 were of death and 10 were of CVD. The causes of death included aortic dissection (*n* = 1), malignancy (*n* = 1), sudden death (*n* = 1), and unknown (*n* = 2). The causes of CVD included ischemic heart disease (*n* = 5), heart failure with complete atrioventricular block (*n* = 1), valvular disease (*n* = 1), peripheral arterial disease (*n* = 2), and aneurysm of large vessels (*n* = 1).

**Table 1:** Characteristics of patients measuring SPP at dialysis initiation

Variable	All (*N* = 104)	Lower SPPmin (*N* = 33)	Higher SPPmin (*N* = 71)	*p*
Age [y.o.]	74 (61, 81)	78 (71, 84)	72 (58, 79)	< .01
Male gender [*n* (%)]	82 (79%)	25 (76%)	57 (80%)	.61
Cause of ESKD [*n* (%)]				.56
DKD	37 (36%)	15 (45%)	22 (31%)	
Nephrosclerosis	24 (23%)	6 (18%)	18 (25%)	
Chronic glomerulonephritis	13 (13%)	4 (12%)	9 (13%)	
Others/unknown	30 (29%)	8 (24%)	22 (31%)	
Smoking history [*n* (%)]	63 (61%)	20(61%)	43(61%)	1.0
Smoking classification [*n* (%)]				.21
Current smoker	6 (6%)	0 (0%)	6 (8%)	
Ex-smoker	57 (55%)	20 (61%)	37 (52%)	
Never smoker	41 (39%)	13 (39%)	28 (39%)	
Diabetes history [*n* (%)]	42 (40%)	16 (48%)	26 (37%)	.29
CVD history [*n* (%)]	38 (36%)	13 (39%)	25 (35%)	.83
Body mass index [kg/m^2^]	22.8 (20.1, 25.5)	21.0 (19.9, 24.6)	23.0 (20.4, 25.6)	.19
Systolic blood pressure [mmHg]	154 ± 21	153 ± 22	154 ± 21	.70
Diastolic blood pressure [mmHg]	83 ± 15	78 ± 14	85 ± 14	.03
Anti-platelets [*n* (%)]	28 (27%)	13 (39%)	15 (21%)	.06
ACE-i/ARBs [*n* (%)]	51 (49%)	19 (58%)	32 (45%)	.29
Statins [*n* (%)]	48 (46%)	14 (42%)	34 (48%)	.68
Hb [g/dl]	9.2 (8.2, 10.2)	9.3 (8.5, 10.5)	9.2 (8.0, 10.2)	.31
Alb [g/dl]	3.3 (3.0, 3.5)	3.3 (3.0, 3.6)	3.3 (3.0, 3.5)	.70
BUN [mg/dl]	92 (76, 109)	83 (65, 107)	93 (77, 109)	.29
Cr [mg/dl]	8.5 (6.6, 10.6)	7.8 (5.7, 8.9)	8.8 (7.1, 11.5)	.01
Corrected Ca [mg/dl]	9.0 (8.5, 9.4)	9.0 (8.7, 9.4)	8.9 (8.5, 9.4)	.98
IP [mg/dl]	6.0 (5.1, 7.4)	5.8 (5.1, 6.6)	6.0 (5.1, 7.7)	.26
UA [mg/dl]	7.2 (5.7, 8.6)	7.2 (5.9, 8.4)	7.2 (5.7, 8.7)	.61
CRP [mg/dl]	0.17 (0.04, 0.76)	0.15 (0.05, 1.99)	0.18 (0.04, 0.63)	.67
T.chol [mg/dl]	154 (138, 179)	149 (129, 176)	158 (142, 185)	.15
GNRI	91.4 (85.8, 99.3)	89.8 (84.4, 99.3)	91.7 (86.2, 99.3)	.49
ADL (Barthel index score)	100 (100, 100)	100 (100, 100)	100 (100, 100)	.68
SPPmin [mmHg]	69 ± 22	43 ± 10	81 ± 14	< .01
Death or CVD within 1 year [*n* (%)]	15 (15%)	10 (30%)	5 (7%)	< .01

Continuous data are presented as mean ± SD or median (IQR): *SPP* skin perfusion pressure, *SPPmin* minimum SPP at 4 locations on both lower extremities, *ESKD* end-stage kidney disease, *DKD* diabetic kidney disease, *CVD* cardiovascular disease, *ACE-i* angiotensin-converting enzyme inhibitor, *ARB* angiotensin II receptor blocker, *Hb* haemoglobin, *Alb* albumin, *BUN* blood urea nitrogen, *Cr* creatinine, *Ca* calcium, *IP* phosphate, *UA* uric acid, *CRP* C-reactive protein, *T.chol* total cholesterol, *GNRI* geriatric nutritional risk index, *ADL* activities of daily living

**Fig. 2:**
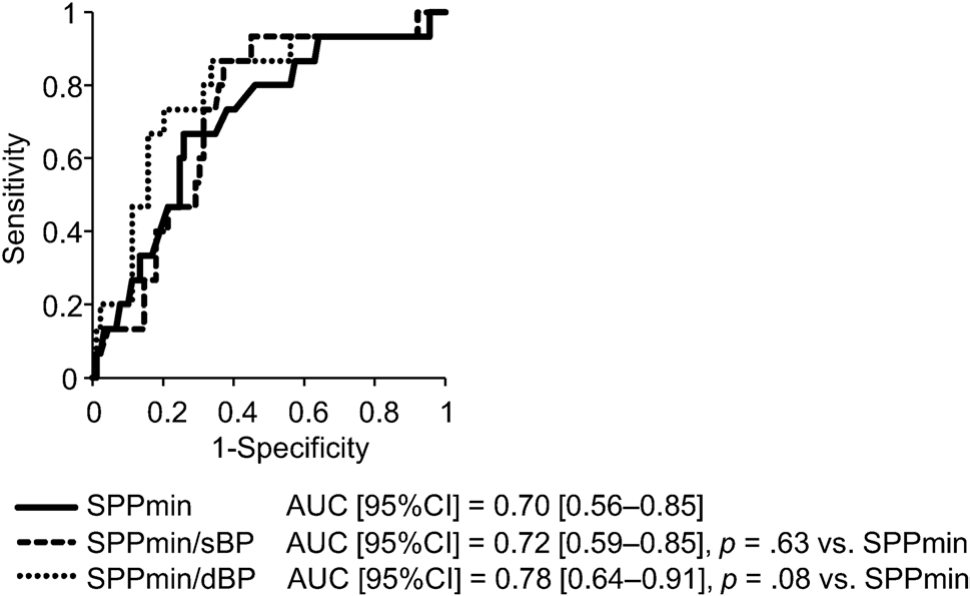
Receiver operating characteristic (ROC) analyses assessing the predictive ability of SPPmin and variables divided by systolic or diastolic pressure for 1-year mortality or CVD after starting HD

Subsequent ROC analysis to elucidate the predictive ability of SPPmin for primary outcome revealed that the cutoff value of SPPmin was 59 mmHg with area under the curve (AUC) [95% confidence interval (95% CI)] of 0.70 [0.56–0.85] (Fig. 2). Therefore, all patients were divided into two groups based on SPPmin greater than or equal to 60 mmHg (higher SPPmin group, *n* = 71) and less than 60 mmHg (lower SPPmin group, *n* = 33) (Table 1). The lower SPPmin group had significantly higher age [78 (71, 84) vs. 72 (58, 79) years old, *p* < 0.01], and incidence of primary outcome (30% vs. 7%, *p* < 0.01) than the higher SPPmin group. By contrast, diastolic blood pressure (78 ± 14 vs. 85 ± 14 mmHg, *p* = 0.03) and Cr [7.8 (5.7, 8.9) vs. 8.8 (7.1, 11.5) mg/dl, *p* = 0.01] were significantly lower in the lower SPPmin group. Findings indicate that ADL was not significantly different between the lower and higher SPPmin groups.

### Relation between SPPmin and clinical factors or outcomes

Results of Spearman’s correlation test indicated a significant correlation between SPPmin and each of age (*r* = − 0.34, *p* < 0.01), diastolic blood pressure (*r* = 0.33, *p* < 0.01), Cr (*r* = 0.39, *p* < 0.01), and T.chol (*r* = 0.22, *p* = 0.04).

**Fig. 3:**
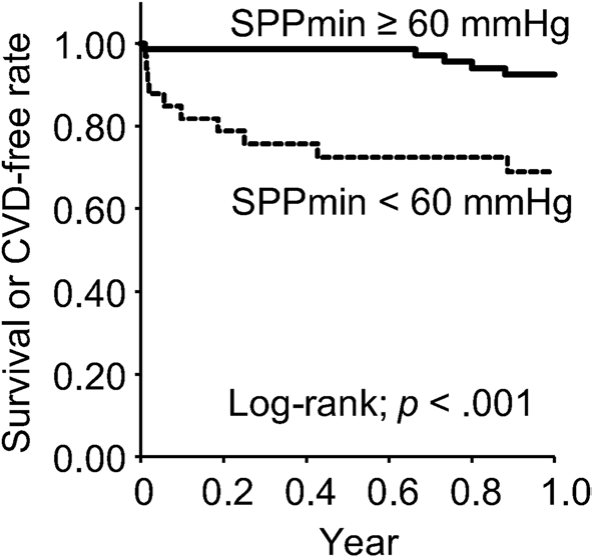
Kaplan–Meier analysis for composite outcome (survival or CVD-free rate) within 1 year after starting HD

Using Kaplan–Meier analysis and log-rank testing, the composite outcome of 1-year mortality or CVD after HD initiation was compared between the lower and higher SPPmin groups (Fig. 3). Results showed that the lower SPPmin group had significantly lower 1-year survival or CVD-free rate (*p* < *0.0*01).

Relations between primary outcome and clinical variables were examined using univariate Cox proportional hazards model analyses. Results showed significant relations between outcome and history of CVD (hazards ratio (HR) [95%CI] = 3.91 [1.33–11.44], *p* = 0.01), SPPmin (HR [95%CI] = 0.97 [0.95–0.99], *p* < 0.01) and lower SPPmin (HR [95%CI] = 5.18 [1.77–15.18], *p* < 0.01) (Table 2). Results of multivariate analysis suggest that lower SPPmin (SPPmin < 60 mmHg) was significantly associated with the composite outcome (Table S1).

**Table 2:** Results of univariate Cox proportional hazards model analyses for 1-year mortality or CVD

Variable	HR (95% CI)	*P*
Age	1.03 (0.99–1.07)	.16
Male gender	3.90 (0.51–29.67)	.19
Smoking history	0.51 (0.19–1.42)	.20
Diabetes history	1.65 (0.60–4.55)	.33
CVD history	3.91 (1.33–11.44)	.01
Body mass index	1.04 (0.93–1.16)	.49
Systolic blood pressure	0.99 (0.97–1.01)	.33
Diastolic blood pressure	1.04 (0.995–1.08)	.08
Anti-platelets	2.65 (0.96–7.32)	.06
ACE-i/ARB	1.64 (0.58–4.60)	.35
Statins	2.45 (0.84–7.18)	.10
Hb	0.95 (0.67–1.34)	.76
Alb	0.52 (0.21–1.30)	.16
BUN	0.99 (0.98–1.01)	.55
Cr	0.92 (0.79–1.07)	.27
Corrected Ca	0.71 (0.39–1.32)	.28
IP	0.82 (0.60–1.12)	.21
UA	0.96 (0.76–1.21)	.74
CRP	1.01 (0.87–1.18)	.86
T.chol	0.99 (0.97–1.01)	.29
GNRI	0.99 (0.95–1.04)	.64
ADL (Barthel index score)	1.00 (0.97–1.03)	.86
SPPmin	0.97 (0.95–0.99)	< .01
Lower SPPmin (< 60 mmHg)	5.18 (1.77–15.18)	< .01

*CVD* cardiovascular disease, *ACE-i* angiotensin-converting enzyme inhibitor, *ARB* angiotensin II receptor blocker, *Hb* haemoglobin, *Alb* albumin, *BUN* blood urea nitrogen, *Cr* creatinine, *Ca* calcium, *IP* phosphate, *UA* uric acid, *CRP* C-reactive protein, *T.chol* total cholesterol, *GNRI* geriatric nutritional risk index, *ADL* activities of daily living, *SPPmin* minimum skin perfusion pressure at 4 locations on both lower extremities

To evaluate whether differences exist in the relation between primary outcome and SPPmin according to the patient background, univariate Cox regression analysis was applied for each subgroup divided by age (≥ 65 years, < 65 years), gender (male), smoking history, diabetes history, and CVD history (Table 3). Results showed a significant relation between lower SPPmin (SPPmin < 60 mmHg) at HD initiation and 1-year death or CVD in the subgroup of patients who were older, male, and with a history of smoking, diabetes, and CVD.

**Table 3:** Results of univariate Cox regression analyses of SPPmin for the primary outcome in subgroups divided by patient background

Subgroup	Incidence of death or CVD [*n*(%)]	HR (95% CI)	*p*
Age			
< 65 (*N* = 35)	3 (9%)	9.42 (0.85–104.30)	.07
> = 65 (*N* = 69)	12 (17%)	3.93 (1.18–13.10)	.02
Gender*			
Male (*N* = 82)	14 (17%)	7.13 (2.23–22.80)	< .01
Smoking history			
No (*N = 41*)	8 (20%)	4.68 (1.11–19.79)	.04
Yes (*N = 63*)	7 (11%)	6.18 (1.20–31.88)	.03
Diabetes history			
No (*N* = 62)	7 (11%)	2.28 (0.51–10.23)	.28
Yes (*N* = 42)	8 (19%)	14.69 (1.80–119.77)	.01
CVD history			
No (*N* = 66)	5 (8%)	1.62 (0.27–9.71)	.60
Yes (*N* = 38)	10 (26%)	12.34 (2.59–58.78)	< .01

^*^Analysis of the female subgroup was omitted because of the small number of cases. *SPPmin* minimum skin perfusion pressure at 4 locations on both lower extremities, *CVD* cardiovascular disease

Because of concerns about the potential effects of systemic blood pressure on SPP measurement [18], we evaluated the relation between SPPmin divided by systolic or diastolic blood pressure (SPPmin/sBP or SPPmin/dBP) and outcome. Results of ROC analysis showed that the predictive abilities of SPPmin/sBP and SPPmin/dBP for the primary outcome were not significantly inferior to that of SPPmin (Fig. 2).

### Identification of PAD by SPP at HD initiation

When the cut-off value of SPP for detection of PAD was set as less than 50 mmHg, 23 of 104 patients (22%) were identified as PAD at HD initiation [4, 19]. Although 6 of 23 patients (26%) with decreased SPP had symptoms such as pain or coldness in the lower extremities, the remaining 17 patients (74%) were asymptomatic.

### Influence of fluid status and hypotension on SPP measurement

Because the influences of dehydration and/or hypotension during the HD session measuring SPP might pose a concern, the amount of dehydration, its ratio to post-dialysis body weight, and the presence of intradialytic hypotension during the HD session in which SPP was measured were evaluated. No data were found to be significantly different between the lower and higher SPPmin groups (Table 4).

**Table 4:** Fluid status change and intradialytic hypotension in the lower and higher SPPmin groups

Variable	Lower SPPmin (*N* = 33)	Higher SPPmin (*N* = 71)	*p*
Ultrafiltration volume during the HD session measuring SPP			
Amount (L)	0.4 (0.2, 1.1)	0.6 (0.3, 1.0)	.68
Ratio of amount to post-dialysis body weight (%)	0.8 (0.1,1.9)	1.0 (0.5, 1.5)	.79
Intradialytic hypotension during HD session measuring SPP [*n* (%)]	2 (6%)	1 (1%)	.24
Weight reduction from HD session measuring SPP to the last one before discharge			
Amount (kg)	1.5 (0.6, 3.3)	1.7 (0.9, 2.6)	.92
Ratio of amount to body weight before discharge (%)	3.2 (1.3, 5.1)	2.6 (1.7, 4.0)	.84

We were also concerned about the effect of the degree of fluid retention on the SPP measurements. When body weight reduction from the HD session measuring SPP to the last one before discharge was evaluated, the amount and its ratio to the body weight before discharge were not found to be significantly different between the lower and higher SPPmin groups, suggesting that the degrees of fluid overload at SPP measurement are comparable between the two groups (Table 4).

## Discussion

This report is the first of a study demonstrating that the lowest value of SPP in bilateral plantar or dorsal foot site at HD initiation is significantly associated with mortality or CVD within one year. The relation between SPPmin and prognosis seemed to hold true for each subgroup of older age or male, and with a history of smoking, diabetes, and CVD.

Several reports in the literature describe investigations of the relationship between SPP and prognosis in dialysis patients. Most were for maintenance HD patients, with measurement of SPP around 1.8 to 17 years after starting HD [11, 13–15]. Considering the strong association between PAD and mortality or CVD and the progression of vascular lesion in dialysis patients [2], examination of blood flow in lower extremities as early as possible, at dialysis initiation, might make sense in terms of assessing prognostic risk and in terms of providing appropriate treatment and care for patients. An earlier cross-sectional study evaluating SPP at HD initiation found that 24.3% of patients already had PAD and that 70% of them were asymptomatic [19]. Results obtained from our study demonstrated that SPPmin at HD initiation was associated with death or CVD within 1 year. We also showed that PAD, defined as SPPmin less than 50 mmHg at HD initiation, was identified in 22% of patients and that 74% of them were asymptomatic. Our findings suggest SPP measurement at HD initiation as a useful tool not only to allow early prognostic risk assessment but also to prevent “missing” cases of impaired lower-limb blood flow, which are asymptomatic.

Results from Kaplan–Meier analysis and the Cox proportional hazards model showed that SPPmin less than 60 mmHg at HD initiation is associated significantly with 1-year adverse outcome in this study. Several reports have suggested cut-off values of SPP for the prediction of prognosis in maintenance HD patients [11, 13–15]. Hiratsuka et al. reported that the optimal cut-off value of SPP for predicting new cardiovascular events or death within a median follow-up of 4.2 years is approximately 70 mmHg [15]. Based on ROC analysis, Otani et al. reported that a cut-off point of SPP for the prediction of mortality within a mean follow-up of 3.2 years was 54.0 mmHg, with a sensitivity of 55% and specificity of 84% [14]. Hatakeyama et al. showed that < 70 mmHg of SPP is associated with poor lower-limb and patient survival within 2 years [13]. From cross-sectional studies, cut-off values of SPP in relation to the presence of PAD or atherothrombotic disease were inferred as 50 mmHg or 53 mmHg in dialysis patients [4, 20]. Our SPP value of less than 60 mmHg for poor prognosis in incident HD patients was close to values reported from other studies, as described above.

Results of the present study suggest that the significant relation between decreased SPP and poor prognosis observed in the overall cohort might also hold true for each subgroup with older age, male gender, diabetes, smoking history, and history of CVD. This basic patient information used for subgrouping includes risk factors not only of CVD or mortality but also of PAD in ESKD patients [13, 21, 22]. Our results suggest that SPP measurements should be taken aggressively to assess prognostic risk, especially when basic clinical information already indicates the patient as being at high risk for CVD or PAD.

A possible effect of pre-existing diseases before the HD initiation on the outcome might be a point of concern. Before starting HD, although no patient had any advanced malignancy, three patients in the lower SPPmin group and one in the higher SPPmin group had been identified already as having untreated CVDs. Even after excluding these cases, the occurrence of outcomes within 1 year after starting HD was still higher in the lower SPPmin group than in the higher SPPmin group (23% vs. 6%, *p* = 0.02). At our facility, screening tests for CVD before HD initiation could not be performed uniformly for all patients, except for echocardiography to consider vascular access. However, detailed interviews and physical examinations during inpatient dialysis initiation might facilitate the identification and determination of CVD treatment strategies. Indeed, all patients were initiated with HD under inpatient conditions: five patients in the lower SPPmin group and one in the higher SPPmin group were identified as having CVDs during hospitalization. They decided on treatment in consultation with specialists, irrespective of the presence or absence of symptoms. Therefore, dialysis initiation under hospitalization might have some importance as an opportunity to screen CVDs. Moreover, considering the results of our study, measuring SPP at dialysis initiation might make an additional contribution to the active search for CVDs.

From this study, a significant positive correlation was found between SPP and dBP. Similar results have been described in an earlier report [18]. When systemic blood pressure is reduced, adequate blood flow would not be delivered to the stenotic arteries and tissue beyond, thereby resulting in a low SPP value [18, 23]. Therefore, we obtained variables by dividing SPPmin by systemic blood pressure and then examined whether these variables had different predictive capability for primary outcomes from SPPmin (Fig. 2). ROC analysis showed that SPP and variables of SPP divided by blood pressure (SPPmin/sBP and SPPmin/dBP) were equivalent in their ability to predict a primary outcome. Consequently, the blood pressure effects on SPP did not appear to be sufficiently large to change the relation between SPP at HD initiation and prognosis.

This study demonstrated that SPP measured during HD session was significantly associated with prognosis. The timing of SPP measurements in maintenance HD patients varied among reports, but most were taken during or after an HD session [4, 11, 13, 15, 18, 20, 24]. An earlier study showed that the SPP value measured on an HD day had higher predictive ability for wound healing than that on a non-HD day in patients with chronic limb-threatening ischemia [18]. Blood pressure fluctuations during and after haemodialysis, especially hypotension, might lead to lower SPP values. Considering reports demonstrating that intradialytic hypotension is associated with PAD or chronic limb-threatening ischemia, it might be reasonable to take SPP measurements during or immediately after HD in light of their sensitive detection of lower extremity blood flow impairment [23, 25]. When dialysis nurses perform SPP during HD at our facility, they might be able to understand the patient's condition more comprehensively and be able to provide care for the patient more appropriately. Therefore, measurement of SPP during an HD session seems worthy of consideration.

For maintenance haemodialysis patients, smoking has been known as one risk factor of PAD [21, 26]. Earlier studies showed a higher rate of current smokers as associated with lower SPP levels [13]. However, smoking status was not found to be significantly different between the lower and higher SPPmin groups in our study. Possible reasons why the relation between smoking and SPP in our study differs from that in earlier reports are the small proportion of current smokers in our cohort (6% vs. 15%) and the fact that we included incident haemodialysis patients. Further studies must be conducted to evaluate the influence of smoking on SPP at dialysis initiation.

Our study had several limitations. First, because this study was conducted at a single centre with a small number of patients, it remains unclear whether the results are applicable to patients of other hospitals. Second, ABI was not performed for the patients. We were unable to compare the predictive capability of SPP and ABI. Third, overfitting was a concern when conducting multivariate analysis because of the small number of primary outcomes. To address these limitations, a large, multicentre cohort study should be conducted.

In conclusion, this study demonstrated that lower SPP in lower extremities at HD initiation is associated significantly with 1-year mortality or development of CVD. Measurement of SPP might be useful for prognostic assessment, even in patients with CVD risk factors. Additional large studies must be conducted to reinforce the results found from this study and to identify effective interventions or care to improve prognosis based on SPP at HD initiation.

## Supplementary Information

Below is the link to the electronic supplementary material.

Supplementary file1 (DOCX 31 KB)

## Data Availability

The data that support the findings of this study are available from the corresponding author, [Y.H.] upon reasonable request.
