# Supplementary material for: Skin perfusion pressure in lower extremities at haemodialysis initiation is associated with 1-year mortality and cardiovascular disease
Source: Clin Exp Nephrol. 2025 Apr 23;29(9):1262–70. doi: 10.1007/s10157-025-02680-1 (PMC12441104; doi:10.1007/s10157-025-02680-1)
Supplement: Supplementary file 1 — Supplementary file1 (DOCX 31 KB) [file 10157_2025_2680_MOESM1_ESM.docx]

Table S1. Results from multivariate Cox proportional hazards model for composite primary outcome

| **Variable** | **Model 1** | | **Model 2** | | **Model 3** | | **Model 4** | | **Model 5** | |
| --- | --- | --- | --- | --- | --- | --- | --- | --- | --- | --- |
|  | **HR (95% CI)** | ***P*** | **HR (95% CI)** | ***p*** | **HR (95% CI)** | ***p*** | **HR (95% CI)** | ***p*** | **HR (95% CI)** | ***p*** |
| SPPmin < 60 mmHg | 4.62 (1.51–14.17) | < .01 | 5.84 (1.98–17.27) | < .01 | 5.87 (1.87–18.43) | < .01 | 9.16 (2.88–29.16) | < .01 | 6.31 (2.10–18.97) | < .01 |
| age | 1.01 (0.97–1.06) | .53 |  |  | 1.00 (0.96–1.04) | .98 |  |  |  |  |
| CVD history |  |  | 4.48 (1.52–13.20) | < .01 | 4.49 (1.48–13.63) | < .01 |  |  | 4.61 (1.55–13.68) | < .01 |
| smoking history |  |  |  |  |  |  | 0.52 (0.18–1.47) | .21 |  |  |
| diastolic blood pressure |  |  |  |  |  |  | 1.06 (1.02–1.11) | < .01 |  |  |
| Alb |  |  |  |  |  |  |  |  | 0.46 (0.17–1.21) | .11 |

SPPmin, minimum SPP value of four data obtained at bilateral lower extremities; CVD, cardiovascular disease; Alb, albumin.
